# Bibliometric assessment of world scholars’ international publications related to conceptual metaphor

**DOI:** 10.3389/fpsyg.2022.1071121

**Published:** 2022-11-22

**Authors:** Ying Han, Zhibin Peng, Hong Chen

**Affiliations:** ^1^School of European Language and Culture Studies, Dalian University of Foreign Languages, Dalian, China; ^2^Foreign Language Research Department, Beijing Foreign Studies University, Beijing, China; ^3^Shanghai International College of Intellectual Property, Tongji University, Shanghai, China

**Keywords:** Conceptual Metaphor Theory, hot topics, bibliometric analysis, CiteSpace, research trend

## Abstract

Research on metaphor has gained increasing attention of world’s scholars since the publication of Lakoff and Johnson’s collaborated book *Metaphors We Live By* in 1980. The present study comprises a pioneering review of publications on Conceptual Metaphor Theory (CMT). It aimed to use the CiteSpace software to provide a clear overview of international research in relation to CMT. In total, 4,458 bibliometric recordings ranging from 1980 to 2022 were collected from the Web of Science (WOS) Core Collection. The descriptive analysis presents the trend of annual publications, the top 10 most prolific journals and the top 10 most productive authors. A document co-citation analysis was conducted via CiteSpace to navigate the key documents in this field. A visualization of keywords and its cluster analysis were conducted to show the research fields and dominant topics. The top 5 keywords with high frequency were language, comprehension, conceptual metaphor, discourse, and figurative language. The most prominent 5 clusters are labeled as right hemisphere, self, time, teacher education, and corpus linguistics. The present review through CiteSpace flags the need for more investigations of CMT from more aspects or interdisciplinary studies, such as metaphor translation, metaphor in literature, metaphor and corpus linguistics, etc.

## Introduction

Traditionally, metaphor was considered as a kind of rhetorical flourish that deviates from ordinary language. Moreover, metaphor was viewed as a matter of language alone, not a matter of thought or action. In 1980, Lakoff and Johnson challenged the traditional view of metaphor in their seminal book *Metaphors We Live By*, arguing that metaphor is ubiquitous in everyday life, not just in language, but also in thought and action, that is to say, our conceptual system is fundamentally metaphorical (Lakoff and Johnson, 1980, p. 3). Our conceptual system plays a key role in defining our everyday realities. If our conceptual system is metaphorical, then what we do, what we experience and the way we think in daily life, is mainly a matter of metaphor. Therefore, metaphor is very important in our everyday life, thus we cannot live without metaphor, as the title of the book suggests that metaphors are the things we live by.

In order to differentiate it from traditional view of metaphor, the metaphor theory proposed by Lakoff and Johnson (1980) is called Conceptual Metaphor Theory (CMT for short). The view that our conceptual system is metaphorical has aroused a large number of scholars’ interest in metaphor research from different disciplines since 1980. Based on the view that abstract concepts are largely metaphorical, Lakoff and Johnson (1999) proposed Embodied Philosophy, which challenges the western philosophical tradition and is considered as the philosophical foundation of Cognitive Linguistics. Besides linguistics, CMT has had great impact on other disciplines. International publications concerning CMT have proliferated across disciplines, such as linguistics, cognitive science, philosophy, literature, anthropology, sociology, etc. However, there is no systematic review on metaphor research till now. It seems that a comprehensive review of studies on CMT cross disciplines is in need. Therefore, the present study intends to conduct a bibliometric analysis of articles related to CMT collected in the database of Web of Science Core Collection with the aid of CiteSpace to present the *status quo* in the area of CMT. Specifically, the following questions will be addressed in this study:

Q1: What are the most fruitful journals and who are the most productive authors in the field of CMT research?

Q2: What are the critical articles in the field of CMT research?

Q3: What are the active research areas and the recent trends in CMT research?

## Methods

### Data collection

To understand how CMT research has developed since 1980, the present study collected articles/reviews through an advanced search in the Web of Science (WOS) Core Collection of Thomson Reuters, SSCI, and A&HCI sections for the bibliometric analysis. All collected articles/reviews were written in English, and we retrieved the data using the following fields:

1. Title = (“metaphor*”), which means that only articles with “metaphor” in the title will be retrieved.
2. Time span = 1980–2022
3. Document type = article OR review
4. (“*”is a wildcard in WOS that represents any group of characters, including no character. For instance, metaphor* = metaphor, metaphors, and metaphorical, etc. Besides, in this article, the review articles do not include book reviews).

7,708 papers in total were obtained from the database distributed in various WoS categories such as “linguistics,” “psychology,” “neurosciences,” “philosophy,” “communication”and “education.” The present study attempted to focus on CMT, but not on traditional view of metaphor. Therefore, the category of “literature (643)” is excluded since its studies are not from the perspective of CMT, but are considered as a traditional literal device. Finally, only 4,458 documents were obtained, which were collected from WoS categories of “linguistics,” “psychology experimental,” “education research,” “communication,””philosophy,” “religion,” “humanities multidisciplinary,” “psychology multidisciplinary,” “social sciences interdisciplinary,” “neurosciences,” “psychology,” “anthropology,” “sociology,” “art,” “history,” “cultural studies” and “political studies.”

### Instrument

CiteSpace 6.1 R2, a bibliometric analysis program created by Chen (2004), is the technology used in this study (Chen, 2004, 2006, 2017; Chen et al., 2010; Chen and Song, 2019). A quantitative strategy for assessing and analyzing the body of literature in a particular topic is provided by bibliometric analysis (Mou et al., 2019, p. 221; Chen, 2020). As one of the most popular bibliometric tools, CiteSpace is “a Java application for analyzing and visualizing co-citation networks” (Chen, 2004, p. 363). It provides a range of analyses, including reference journal analysis and keyword analysis, to assist academics spot present and upcoming research trends in an area (Mou et al., 2019). Although there is a significant amount of international research on CMT, relatively few studies have used bibliometric techniques to examine this literature. The purpose of this study is to conduct a bibliometric analysis of CMT research from 1980 to 2022 using the CiteSpace software. Visual analytical software called CiteSpace is frequently used to carry out bibliometric analyses (e.g., Ellegaard and Wallin, 2015; Peng and Hu, 2022).

A collection of bibliographic data files in the field-tagged Institute for Scientific Information Export Format constitute the software’s input. The files used in this investigation are taken from the WoS core collection. We select “full record and cited references” as the record content, and CiteSpace tool can directly identify the files. The following procedural processes will be carried out on the files once they are loaded into the software: time slicing, thresholding, modeling, pruning, merging, and mapping (for more information, see Chen, 2004). Cocitation networks are represented as the software’s outputs, and each network is shown in its own interactive window interface. It can illustrate the overall state of a particular field, show the evolution of a knowledge field on a citation network, and highlight some important documents in the development of a field. The analysis and visualization of thematic structures and research hotspots is where CiteSpace excels. It can give us networks of co-citations between references, authors, countries, etc.

Co-citation documents and keyword co-occurring analysis were carried out in this study using this software to find significant references, identify research trends, and identify research hotspots in the international research on CMT.

## Results

### Publication years, journals, and productive authors on metaphor

**FIGURE 1:**
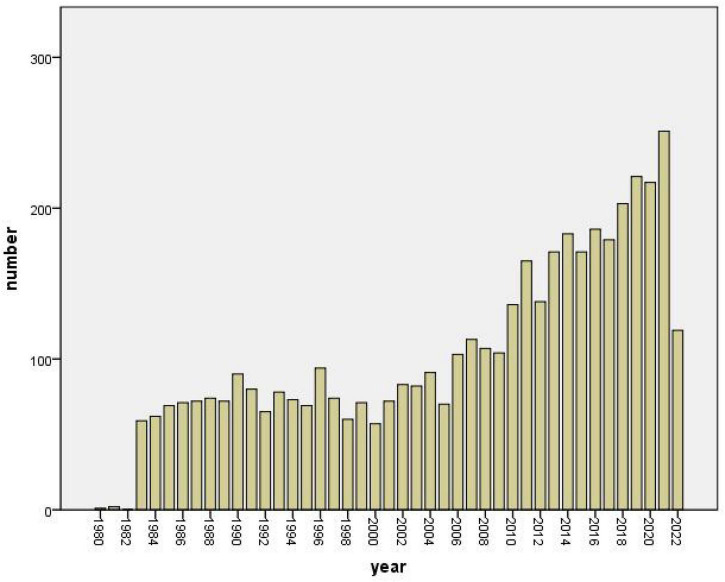
Annual publication on metaphor in WoS.

In total, 4,458 articles and reviews about CMT research met the criteria. Figure 1 shows the number of articles published annually on metaphor since 1980. There is only 1 publication in 1980, 2 publications in 1981, and no publication in 1982. In 1983, there is a huge surge with 59 publications on metaphor. After 1983, there is a continued growth in general, and in 2021, the number of articles pertaining to metaphor reaches the peak with 251 publications.

Table 1 presents the top 10 most fruitful journals for metaphor study. *Metaphor and Symbol*, as the only SSCI journal specializing in metaphor study, ranked the top in the number of published articles, with 304 publications related to metaphor. *Journal of Pragmatics* ranked the second in the number of published articles, with 98 publications about metaphor, followed by *Semiotica* and *Frontiers in Psychology*, with 70 and 65 publications on metaphor respectively. *Review of Cognitive Linguistics* and *Cognitive Linguistics* are journals which only publish topics related to the linguistic school of Cognitive Linguistics, and metaphor is one of the most studied sub-fields in Cognitive Linguistics, so *Review of Cognitive Linguistics* (47) and *Cognitive Linguistics* (41) ranked the fifth and sixth in the rankings. They did not rank the top three because they only publish about 20 papers each year. Metaphor researchers can refer to Table 1 to find suitable journals as their papers’ outlets when they are considering a submission.

**TABLE 1:** Top 10 most prolific journals for metaphor study.

Ranking	Journals	The number of published papers
1	*Metaphor and Symbol*	304
2	*Journal of Pragmatics*	98
3	*Semiotica*	70
4	*Frontiers in Psychology*	65
5	*Review of Cognitive Linguistics*	*47*
6	*Cognitive Linguistics*	41
7	*Journal of Psycholinguistic Research*	37
8	*Brain and Language*	*32*
9	*Discourse Society*	27
9	*IBERICA*	27

Table 2 presents the top 10 most productive authors for metaphor study. Gibbs ranked the top in the number of papers published on metaphor (33), followed by Faust (24) and Steen (23). Steen is the pioneer of Deliberate Metaphor Theory (Steen, 2010, 2015). Among the top ten most productive authors, Faust, Mashal (top 4, 22), and Katz (top 5, 18) are all mainly focusing on the cognitive processing of metaphor.

**TABLE 2:** Top 10 most productive authors for metaphor study.

Ranking	Authors	The number of published papers
1	Gibbs, W.	33
2	Faust, M.	24
3	Steen, J.	23
4	Mashal, N.	22
5	Katz, N.	18
6	Semino, E.	16
7	Glucksberg, S.	15
7	Littlemore, J.	15
7	Ritchie, D.	15
10	Burgers, C.	14
10	Kennedy, M.	14

### Document co-citation analysis

**FIGURE 2:**
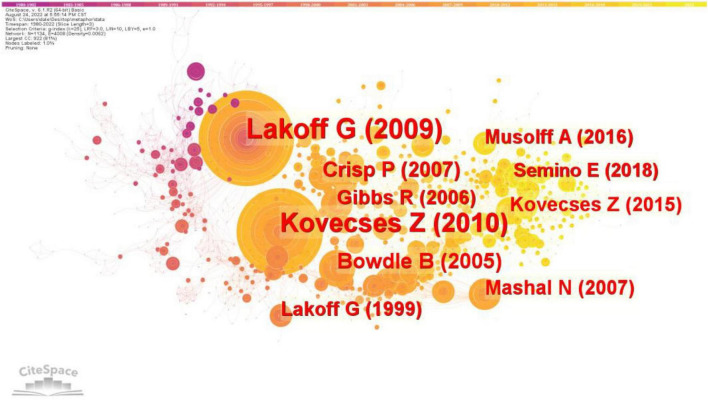
Critical publications in metaphor research.

The citation frequency of literature is a basic index for academic circles to evaluate the influence of literature. The higher the citation frequency is, the higher the application value and reference value of a literature are, and its research methods or conclusions have exerted an important influence in this field. Document co-citation analysis via CiteSpace can help us find out the key documents in a knowledge domain. We conducted a document co-citation analysis of the 4,458 documents collected from the WoS. The 4,458 bibliographic recordings from 1980 to 2022 were visualized. We chose a three-year time slice and selected the top 50 most cited articles in every 3 years. The results are illustrated in Figure 2. There were 1,134 individual nodes and 4,008 links, representing cited articles and co-citation relationships among the whole data set, respectively. Table 3 presents the top 10 most cited publications in the field of metaphor study.

**TABLE 3:** The top 10 most cited publications in metaphor study.

Citation count	Author (year)	Publication name	Journal or press
131	Lakoff and Johnson (1980)	*Metaphors We Live By*	Chicago: University of Chicago Press
104	Kövecses (2010)	*Metaphor: A practical introduction* (2nd ed.)	Oxford University Press
41	Bowdle and Gentner (2005)	The Career of Metaphor	*Psychological Review*
39	Pragglejaz Group^1^ (2007)	MIP: A Method for Identifying Metaphorically Used Words in Discourse	*Metaphor and Symbol*
31	Gibbs (2006)	*Embodiment and Cognitive Science*	Cambridge University Press
31	Kövecses (2015)	*Where Metaphors Come from: Reconsidering Context in Metaphor*	Oxford University Press
30	Mashal et al. (2007)	An fMRI investigation of the neural correlates underlying the processing of novel metaphoric expressions	*Brain Lang.*
29	Lakoff and Johnson (1999)	*Philosophy in the Flesh: the Embodied Mind & its Challenge to Western Thought*	Chicago: University of Chicago Press
27	Musolff (2016)	*Political Metaphor Analysis: Discourse and Scenarios*	Bloomsbury Academic
26	Semino et al. (2018)	An integrated approach to metaphor and framing in cognition, discourse, and practice, with an application to metaphors for cancer	*Applied Linguistics*

^1^The original members of Pragglejaz were Peter Crisp (Chinese University of Hong Kong), Raymond Gibbs (University of California, Santa Cruz), Alice Deignan (University of Leeds), Graham Low (University of York), Gerard Steen (Vrije University of Amsterdam), Lynne Cameron (University of Leeds/The Open University), Elena Semino (Lancaster University), Joe Grady (Cultural Logics), Alan Cienki (Emory University), and Zoltan Kövecses (Eötvös Loránd University).

From 1980 to 2022, a total of 27 documents were cited for more than 20 times. The most cited document is a book written by Lakoff and Johnson (1980). It is cited most because it’s a seminal book which changes our understanding of metaphor. It argues that metaphor is not only a rhetoric device, but importantly, it is a way of thinking. In a word, our conceptual system is fundamentally metaphorical. The work of Lakoff and Johnson (1980) serves as the foundation for the cognitive linguistic approach to metaphor. It draws attention to the conceptual, as opposed to purely linguistic character of metaphor. A “mapping” from a “source (conceptual) domain” to a “target domain” is how it defines metaphor. So a statement like “I defended my argument” can be explained by a conceptual metaphor ARGUMENT IS WAR, where “argument” is the target and “war” is the source. Conceptual Metaphor Theory (CMT) is an influential theory in Cognitive Linguistics and publications about CMT have proliferated since 1980.

The second most cited document is a monograph by Kövecses (2010). It is an introduction to CMT. It provides an overview of what has happened in the past three decades in the cognitive linguistic study of metaphor since 1980. It is easy to read and it can help those who are not familiar with CMT to get a quick overview of the current state of metaphor theory.

Bowdle and Gentner (2005)’s article is the third most cited one. How metaphors establish mappings between concepts from different domains is a key issue in metaphor study. The structure-mapping theory is used by the authors to propose an evolutionary path. This hypothesis—the career of metaphor—postulates a shift in mode of mapping from comparison to categorization as metaphors are conventionalized. The language that people employ to make figurative assertions also reflects this processing shift, as shown by three experiments. The career of metaphor hypothesis offers a unified theoretical framework that can resolve the debate between comparison and categorization models of metaphor. According to this account, the degree of conventionality and linguistic form of a metaphor will determine whether it is processed directly or indirectly, whether it operates at the level of an individual concept or at the level of a complete conceptual domain.

The fourth most cited publication was written by Pragglejaz Group (2007) about how to identify metaphorically used words in discourse. For metaphor researchers, how to identify and explicate metaphoric language in real discourse is a key issue. This article provides a practical Metaphor Identification Procedure (MIP) for metaphor researchers to follow. Its general purpose is “to provide a research tool that is relatively simple to use and flexible for adaptation by scholars interested in the metaphorical content of realistic discourse” (Pragglejaz Group, 2007, p. 2). To date, MIP is the most widely used metaphor identification tool. Most of the metaphor scholars used MIP to find metaphorically used words in discourse.

Gibbs’ (2006) monograph ranked the fifth most cited publication on metaphor. This book examines how some of the fundamental underpinnings of human cognition and language are derived from people’s subjective, felt perceptions of their bodies in motion. It is important to study cognition in terms of the dynamic interactions between people and their environment because cognition is what happens when the body interacts with the physical and cultural world. Human mind and language develop from recurrent embodied activity patterns that limit on-going intelligent conduct. We must look for the broad and specific ways that language and mind are intimately impacted by embodied action rather than assuming that cognition is entirely internal, symbolic, algorithmic, and disembodied. The wealth of empirical evidence that supports the notion that the mind is embodied is described by Embodiment and Cognitive Science. This evidence comes from numerous disciplines and includes work on perception, concepts, imagery and reasoning, language and communication, cognitive development, emotions and consciousness.

Kovecses’ (2015) monograph is also the fifth most cited one with 31 citations. This book makes the case that “Conceptual Metaphor Theory” has proven to be effective throughout time. At the same time, it also implies that the idea of conceptual metaphor, according to which conceptual metaphors are based on our bodily experience, is enhanced and refined by the notion of metaphorical grounding put forth in the book. The body can thus be seen as just one of the many contexts from which metaphors can emerge (including the situational, discourse, and conceptual-cognitive contexts), even though it may be the dominant or crucial one. This is true in many instances of metaphor, but the role of the body in metaphor creation can and should be reinterpreted. Such a proposal appears to be more in accord with what has recently been learned about the nature of human cognition, namely that human cognition is anchored in experience in many different ways, with embodiment, strictly speaking, being just one of them. According to the research presented here, this is due to the fact that cognition, including metaphorical cognition, is rooted in a variety of contexts, including the situations in which people act and live, the discourses in which they are constantly interacting and communicating with one another, and the conceptual knowledge they have gained about the world through their experiences there.

### Co-occurring terms analysis

In a sense, keywords express an article’s main idea and subject matter and act as its central summary. When two keywords appear together in a piece of writing, it shows how closely related they are and how strong their association is. The general consensus is that the more closely connected two or more terms are, the more frequently they will appear together. To define the strength, CiteSpace offers a function called Betweenness Centrality. In other words, it is likely that we will see a keyword even if we discuss other related topics if it frequently appears alongside other different phrases. Therefore, the higher the Betweenness Centrality value this keyword exhibits, the status of the keyword can be more important. The research fields and dominant topics were identified in this study using a keyword co-occurrence analysis.

**FIGURE 3:**
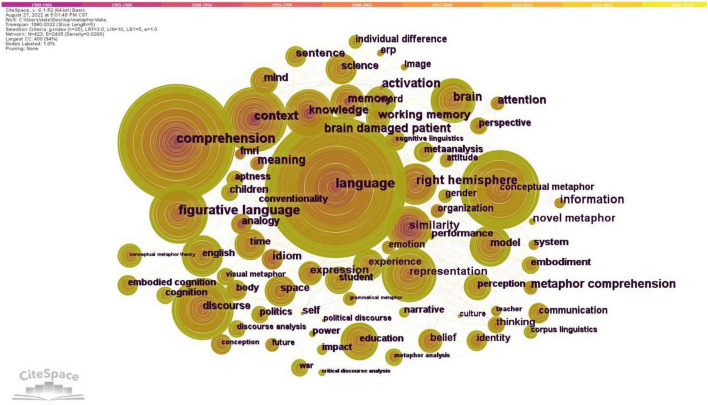
Keyword co-occurrence network for documents of metaphor research.

In order to pinpoint the terms or phrases that appeared in at least two different publications, a term analysis of words derived from keywords was carried out. High frequency terms may be used to identify hotspots in a particular research area (Chen, 2004). Language, comprehension, conceptual metaphor, discourse, and figurative language made up the top 5 keywords with high frequency. Figure 3 depicts the network of related keywords, and Table 4 lists the terms with a frequency of greater than 20.

**TABLE 4:** Co-occurring terms with high frequency.

Count	Central	Keyword	Count	Central	Keyword	Count	Central	Keyword
294	0.10	Language	41	0.00	Aptness	27	0.02	Embodiment
187	0.06	Comprehension	41	0.04	Belief	27	0.00	Future
152	0.01	Conceptual metaphor	40	0.05	Politics	27	0.02	Metaphor comprehension
98	0.04	Discourse	39	0.01	Conceptual metaphor theory	26	0.03	System
92	0.06	Figurative language	38	0.03	Analogy	26	0.01	Individual difference
85	0.07	Context	38	0.03	Communication	25	0.03	Attention
83	0.05	Representation	38	0.01	Cognitive linguistics	25	0.01	Corpus linguistics
74	0.01	Model	36	0.03	Thinking	25	0.05	Activation
71	0.03	Right hemisphere	35	0.04	Gender	25	0.01	Cognition
70	0.02	Similarity	35	0.01	Metaanalysis	24	0.02	Emotion
67	0.02	English	33	0.03	Children	24	0.01	Political discourse
67	0.07	Knowledge	33	0.01	Embodied cognition	24	0.00	Critical discourse analysis
66	0.04	Brain	32	0.03	Perspective	24	0.02	Brain damaged patient
64	0.05	Science	32	0.00	Metaphor analysis	24	0.02	Organization
59	0.01	Education	31	0.04	Meaning	24	0.01	fMRI
51	0.03	Memory	31	0.01	Visual metaphor	23	0.00	Grammatical metaphor
51	0.04	Expression	31	0.02	Identity	23	0.03	Performance
50	0.02	Working memory	30	0.01	War	23	0.00	Conception
50	0.02	Perception	30	0.02	Body	22	0.01	Power
49	0.01	Time	28	0.02	Sentence	22	0.02	Image
47	0.02	Space	27	0.06	Self	21	0.00	Teacher
47	0.03	Mind	27	0.01	Discourse analysis	21	0.01	Impact
45	0.04	Student	27	0.01	Culture	20	0.02	Novel metaphor
45	0.04	Idiom	27	0.01	Discourse analysis	20	0.04	Narrative
43	0.08	Information	27	0.01	Culture	20	0.01	Conventionality
42	0.03	Experience	27	0.01	Attitude	20	0.01	ERP
42	0.01	Word						

### Cluster interpretations

**FIGURE 4:**
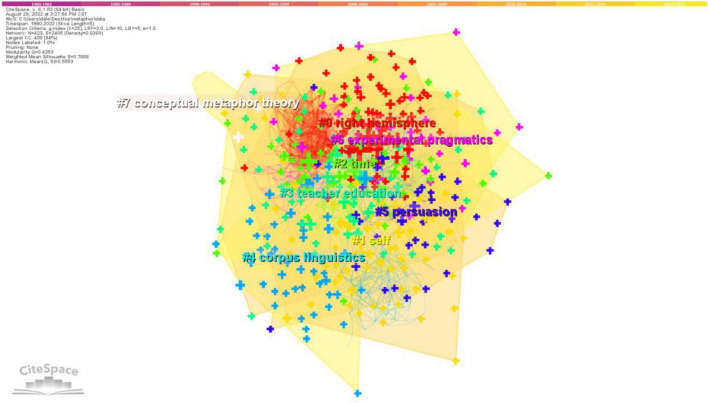
Cluster view of keyword co-occurrence for metaphor study.

A cluster analysis was conducted based on the keyword co-occurrence analysis. The 4,458 bibliometric recordings generated 27 clusters in total. Figure 4 shows 8 most important keyword clusters obtained by keyword co-occurrence analysis by Labeling clusters with indexing terms and showing clusters by log likelihood ratio (LLR). Table 5 shows the keywords lists of the seven important clusters in metaphor study. It illustrates an aggregated distribution in which most colorful areas overlapped, indicating that these clusters share some basic concepts or information (as suggested by Chen, 2004).

**TABLE 5:** Important clusters of keywords in metaphor research.

Cluster ID	Size	Silhouette	Cluster names (LLR)	Top terms (LSI)	Top terms (LLR, p-level)
0	84	0.842	Right hemisphere	Figurative language; working memory; conceptual metaphor; language processing; predicative metaphors; modality; coherence; structural alignment	Right hemisphere (76.36,1.0E-4); n400 (47.97, 1.0E-4); fMRI (35.91.1.0E-4); figurative language (28.141.0E-4); novel metaphors (24.381.0E-4)
1	71	0.735	Self	Metaphor; irrationality; change; disorder; absurdity; narrative; discourse; gender; pregnancy; conceptual metaphors	Self (33.1,1.0E-4); gender (19.33, 1.0E-4); infertility (19.33, 1.0E-4); experience (18.9, 1.0E-4); women (16.52, 1.0E-4)
2	67	0. 624	Time	Conceptual metaphor; semantic approximations; cognitive operations; conceptual complexes; first language acquisition; language; implicit; future; gesture; construal;	Time (51.41, 1.0E-4); embodied cognition (47.99, 1.0E-4); conceptual metaphor (44.99, 1.0E-4): space (34.07, 1.0E-4): embodiment (22.99, 1.0E-4)
3	55	0.769	Teacher education	Knowledge; model; representation; text; Chinese learners; teacher beliefs; pre-service science teacher; rasch Measurement; item response theory	Teacher education (39.33, 1.0E-4): science (33.84, 1.0E-4); knowledge (31.78, 1. 0E-4); student (31.44, 1.0E-4); model (31.41, 1.0E-4)
4	51	0.824	Corpus linguistics	Discourse analysis; critical discourse analysis; dialectical-relational approach; hybrid space; science learning; corpus linguistics; social exclusion; social democracy; sound images; radio storytelling	Corpus linguistics (45.44, 1.0E-4): political discourse (40.23, 1.0E-4); discourse analysis (32.51, 1.0E-4); migration (28.12, 1.0E-4); critical discourse analysis (26.16, 1.0E-4)
5	36	0.795	Persuasion	Information persuasion; recall; news; increase; expression; comprehension; dimension; figurative language; analogical transfer;	Persuasion (28.95, 1.0E-4); rhetorical figure (19.85, 1.0E-4); visual metaphor (19.36, 1.0E-4); information (17.89, 1.0E-4); picture (14.88, 0.001)
6	31	0.776	Experimental pragmatics	Figurative language: alzheimers disease: language comprehension: language; working memory; cognitive linguistics, relevance theory; older; elicited speech production; internal state language;	Experimental pragmatics (19.13, 1.0E-4); cognitive linguistics (16.46.1.0E-4): williams syndrome (13.83.0.001): relevance theory (13.83.0.001): pragmatics (13.59.0.001)
7	5	0.991	Conceptual metaphor theory	Conceptual metaphor theory; metaphor clusters; political argumentation; mixed metaphor; visual spatial attention multimodal metaphor; primary metaphors; economic discourse; corpus linguistics	Conceptual metaphor Theory (70.48,1.0E-4); multimodal metaphor (23.44,1.0E-4); political cartoons (15.6, 1.0E-4); social semiotics (15.6, 1.0E-4): visual metaphor (10.3, 0.005)

**Cluster #0 is labeled as right hemisphere.** Processing of metaphoric language is a very important research topic in neurolinguistics. Hemispheric processing of metaphors has been extensively explored by scholars in neurolinguistics and psycholinguistics during the past two decades. It is suggested by many researchers that the right hemisphere (RH) may contribute uniquely to the processing of metaphoric language.

According to behavioral studies using the divided visual field (DVF) paradigm (Faust and Mashal, 2007; Mashal and Faust, 2008) and neuroimaging data (Mashal et al., 2007; Bohrn et al., 2012), left-lateralized processing is more important for processing familiar, traditional metaphors (e.g., broken heart) than unfamiliar, novel metaphors (e.g., mercy blanket). Additionally, conventional metaphors were found to undergo bilateral processing (Bambini et al., 2011; Diaz et al., 2011). The graded salience hypothesis (GSH; Giora, 1997), which states that the level of semantic salience impacts metaphor processing, is typically supported by these findings. Meanings that are more conventional, frequent, familiar, prototypical, and context-independent are easier to obtain than meanings that are less prominent. The figurative meaning of traditional metaphors is more obvious and more approachable than the literal interpretation. In contrast, the literal meaning is more obvious in novel metaphors, while the figurative meaning is only revealed later with context. According to the GSH, the important metaphorical meaning of traditional metaphors is stored in the mental lexicon as opposed to novel metaphors, whose meaning must be acquired through a process of integration and inference. The GSH also predicts a stronger participation of the right hemisphere (RH) in understanding novel and non-salient metaphorical meanings and a greater involvement of the left hemisphere (LH) in understanding traditional and salient metaphorical meanings (Giora, 2003).

Recently, scholars begin to explore the differences between hemispheric processing of metaphoric expressions in a native language versus a second language. An experiment study conducted by Ershaid et al. (2022) shows that a left-hemisphere advantage or a bilateral pattern of processing was observed for conventional metaphors in both varieties of Arabic and in Hebrew, a pair of typologically close languages, suggesting similar hemispheric processing in native and second language.

**Cluster #1 is labeled as self.** Metaphors “can cast light on why we understand ourselves as we do” (Potter and Wetherell, 1987, p. 108). In Garrett’s (1996) words, “metaphors help create the self” (p. 139). Metaphors, in the words of Garrett (1996), “help build the self” (p. 139). As a result, metaphors can be useful in revealing how language users construct their identities, both as individuals and as engaged members of a social network. For instance, Bates (2015) found that pro-ana participants drew on a core set of self metaphors (self as space, self as weight, perfecting the self, and the social self) that served as effective rhetorical tools for controlling self-description and fostering interactional practices. These findings pave the way for further investigation into the function of metaphors in the interpretation of eating disorders and, more specifically, in the formation of identities within the pro-ana community. With a focus on the use of metaphors to discuss the self, it seeks to deepen and advance our understanding of how identities and selfhood are discursively constructed in pro-ana sites.

Understanding the various tools created and used by disorder members to perform their identities is made possible by the examination of metaphorical language used to talk about the self, which adds a new level to the description of disorder online identities. These studies on self-metaphors are thus part of a wide and expanding body of social psychology research that promotes a psychological understanding of self and identity in the area of discourse practices (Potter and Wetherell, 1987; McGannon and Spence, 2010).

**Cluster #2 is labeled as time.** The representation of time in language is a key issue which has drawn much attention in Cognitive Linguistics. Unlike space, which is “out there” for us to discover, time is something that can not be perceived directly by human beings. If there are no “temporal percepts,” what is time? Cognitive linguists answer this question by holding the view that time has a phenomenological basis (Evans, 2019, p. 97). That is to say, time has a subjectively and psychologically real experience, which is directly, at least in part, perceived at the level of subjective awareness. A large body of research by cognitive linguists showcases that our perception of time is grounded in the experience of our spatio-physical environment. Metaphor researchers contrast and compare the domain of time and the domain of space and argue that time is understood through the domain of space, i.e., the spatialization of time. Space is often recruited to understand concepts for time. We can talk and think about the passage of time as the passage of physical objects because there is a stable knowledge structure in our conceptual system which structures temporal passage in terms of physical passage. The stable knowledge structure is what we call conceptual metaphor (Lakoff and Johnson, 1980, 1999). The concept of FUTURE, for example, is conceptualized in many languages as being metaphorically “located” in front of the observer, while the concept of PAST is conceptualized as being metaphorically“located”behind the observer. However, this is not the case for all languages and the cross-linguistic variations of time conceptualization are widely discussed in metaphor research. In Aymara, an indigenous South American language, the concept of FUTURE is conceptualized as being “located” behind the observer, while the PAST is conceptualized as being “located” in front of the observer (Núnez and Sweetser, 2006).

In recent years, how we conceptualize time in terms of space was also discussed in gesture studies. Cognitive linguists try to find evidence in gesture studies to test the findings they find in the study of metaphor languages. The Cognitive Commitment, a postulate of Cognitive Linguistics, asserts that linguistic patterns and structures are based on broader cognitive principles (Lakoff, 1990). As a result, gesture and spoken language are both “manifestations of the same underlying conceptual system that is the basis for the human expressive ability” (Wilcox and Xavier, 2013, p. 95). Research on metaphors has come under fire for its “linguistic circularity” (Murphy, 1996, p. 184). To examine the suitability of theories and models proposed primarily based on verbal modality, one strategy to prevent it is to look for converging evidence from other modality(ies). From this vantage point, the study of co-verbal gestures can add to our understanding of metaphor studies. It may provide evidence for, or give new insights into, or even challenge the findings about the representation of time solely based on metaphorical language.

**Cluster #3 is labeled as teacher education.** Figurative language is easy for native speakers to understand and use. However, second-language speakers often find it particularly difficult to understand those figurative languages. Littlemore et al. (2011) found that 40% of metaphoric expressions, which were understood effortlessly by native speakers, were not understood by second language learners at a British university. Similarly, second-language learners use less metaphors in speaking and writing than native speakers and their metaphors tend to be incorrect and stylistically inappropriate (Kathpalia and Carmel, 2011). The reason for the difficulty for the second-language speakers’ understanding and production of metaphors may lie in the fact that the figurative meaning of an expression is established within the socio-cultural experience of native speakers. As second-language speakers lack enough experience with the language and the culture, those metaphorical expressions, which are accessible to native speakers, are not necessarily accessible to second-language speakers (Kecskes, 2006). Thus, how to improve second-language learners’ ability to comprehend and produce figurative language is a major concern for second-language teachers. Accordingly, how to improve teachers’ ability to improve students’ metaphor using ability is discussed intensely in metaphor research.

### Implications for future study

During the past four decades, CMT has drawn the attentions of scholars across disciplines. Based on the above document co-citation analysis, co-occurring terms analysis, and cluster interpretations via CiteSpace, this study found that metaphor research has largely focused on cognitive processing of metaphors, the influence of metaphors on self identity, the spatialization of time, and second-language learners’ metaphor ability education. The spatialization of time is the study of metaphor theory itself. Metaphor ability education is one particular strand of applied linguistics, while cognitive processing of metaphors, the influence of metaphors on self identity respectively comprise interdisciplinary studies of language and cognitive science, and metaphor and pathology.

The present state of international publications on CMT reveals that scholars around the world have contributed significantly to literature on CMT from various disciplines. However, there is still a dearth of studies concerning some important aspects, such as metaphor translation, metaphor in literature, metaphor and corpus linguistics, etc.

**Metaphor translation**. Metaphor is ubiquitous in all languages, but it is also influenced by different cultures. Metaphor has a natural and inseparable connection with culture. Metaphors will inevitably reveal cultural attributes, including historical events, literary traditions, philosophy, local customs, food, clothing, housing and transportation, aesthetic concepts, and so on. Therefore, a culture-specific metaphor may not find a counterpart in another language. When a culture-specific metaphor exists, the translation of the metaphorical expressions represents a huge challenge to translators. The success of the translation largely depends on the handling of the language-specific metaphor expressions. Facing this difficulty, scholars have explored metaphor translation strategies for different genres, such as news (Van Poucke and Belikova, 2016), legal documents (Bozovic, 2022), metaphorical idioms (Su, 2021), etc. However, there is no consensus on the strategies for translating metaphors since different scholars proposed different strategies, such as literal translation, paraphrase, translation by other rhetoric devices, translation by substitution, omission, and combined strategies. More extensive and in-depth studies of metaphor translation will enable us to learn more about the complications and mechanism of translation and enhance our understanding of CMT, and even our cognition.

**Metaphor in literature.** According to the traditional view, there is a sharp distinction between literal language and figurative language. While literal language is precise and clear in meaning, figurative language (e.g., metaphor) is imprecise and largely belongs to the domain of literature, such as poets and novelists. To be simple, in traditional view, metaphor, whether in linguistics or philosophy, is only a kind of rhetorical flourish which deserves the attention of literature researchers. However, at the onset of CMT, the traditional view is challenged and changed. Cognitive linguist Raymond Gibbs (1994), through examination of the features that are held to distinguish literal and figurative languages and based on lots of different kinds of psycholinguistic experiments, found that there are no such sharp distinctions between literal and figurative languages. The basic premise of CMT is that novel metaphors (the traditional view of metaphor) and dead metaphors (CMT) have no principled distinction and they are both embodiment of our thinking ways. Following the onset of CMT, scholars paid increasing attention to conceptual metaphor, while neglecting the novel metaphors in literature. Therefore, more scholarly attention should paid to those metaphors in literary contexts and the comparison between novel metaphors and conceptual metaphor can deepen our understanding of CMT and literature works.

**Metaphor and corpus linguistics.** Like CMT, corpus linguistics started to grow quickly at the beginning of 1980s and it is widely used in applied linguistics and related fields (Deignan, 2005). CMT and corpus linguistics share some basic assumptions and are complementary. Like CMT, corpus linguistics is usage-based, as it examines actual language data to find out usage patterns or rules. At the early stage of CMT research, scholars (e.g., Lakoff and Johnson, 1980) utilized metaphorical expressions as examples based on their intuitions, which is considered to be too subjective. Further, it is believed by some scholars that it seems that intuition could not predict the more delicate features of linguistic metaphors. Therefore, corpus data can help metaphor researchers to make less subjective statements about issues such as the existence and frequency of literal and metaphorical senses, detailed aspects of their meanings, and their collocational and syntactic behavior (Deignan, 2005). How to retrieve metaphorically used expressions from the corpus is a key issue in using corpora to research metaphor. There are mainly three possibilities. Firstly, when the corpus is large, the researcher can first intuitively work out lists of linguistic metaphors which realize particular conceptual metaphors, and then use these linguistic metaphors to trawl concordance lines in the corpus. Secondly, the researchers can search linguistic metaphors manually in a small corpus, and then find metaphors in the large corpus (Cameron and Deignan, 2003), or search a small sample of the large corpus by hand (Charteris-Black, 2004). The third way into a corpus is to cooperate with the lexicographers who rely on corpus data and ask them to flag metaphorical uses on their database while they work through concordances of the entire lexicon (Deignan, 2005).

### Strengths and limitations

CiteSpace is a technique used to identify trends in international scientific research. The global CMT research included in this study covers publication outputs, productive authors, popular articles, popular keywords and research gaps. To the best of our knowledge, our study was the first to use CiteSpace to examine a significant amount of data from CMT publications published during the previous 40 years. Instead than just reviewing articles and studies about metaphor (Morsanyi et al., 2020; Ivan, 2021), CiteSpace could find more thorough results. Additionally, we were able to spot emerging trends, author collaboration, and co-occurring terms with high frequency thanks to the bibliometric approach.

Although within its delineated scope, the present study aspired to be as comprehensive as possible, some limitations were unavoidable. One limitation of this study is that it only searched documents in the Web of Science. Other data sources such as Scopus, Google Scholar, Index Medicus, or Microsoft Academic Search were neglected. The document type labeled by publishers is not always accurate. Some publications that WoS described as reviews, for instance, weren’t reviews at all (Yeung, 2021). Another limitation of this study is that only one scientometric tool is employed in this review. Thirdly, some potential publications have recently been published, but these studies could not be frequently cited. And due to obliteration by incorporation, the citation count for some older publications may also be low (Li et al., 2021). Future research should be conducted by using a larger database and performed by different tools.

## Conclusion

From Aristotle (1954), scholars have begun to think about metaphor. In a very long time, metaphor is only seen as “a device of the poetic imagination and the rhetorical flourish-a matter of extraordinary rather than ordinary language” (Lakoff and Johnson, 1980, p. 3). It is only when Lakoff and Johnson (1980) published their seminal book *Metaphors We Live By*, which proposed the Conceptual Metaphor Theory, that the cognitive function of metaphor has gained much scholarly attention. In contrast to the traditional view, Lakoff and Johnson (1980) argue that metaphor plays a central role in thought, i.e., our conceptual system is fundamentally metaphorical, and metaphor is indispensable to both thought and language. Since then, CMT has been explored and developed in a large number of studies in the fields of linguistics, psychology, philosophy, cognitive science, anthropology, and sociology, etc. To date, there are huge publications on CMT. However, there is a lack of bibliometric assessment of world scholars’ international publications related to CMT. To fill this gap, the present study carried out a bibliometric study on the relevant and available literature. In total, 4,458 bibliometric recordings ranging from 1980 to 2022 were collected from the Web of Science (WOS) Core Collection. The descriptive analysis shows that there is a continued growth in the number of publications each year, which means that CMT has aroused increasing interests of scholars from different academic disciplines. *Metaphor and Symbol* ranked the top in the number of published articles, with 304 publications related to metaphor. Gibbs is the top author in the number of papers published on metaphor, followed by Faust and Steen. Then CiteSpace software was adopted to visually analyze those bibliometric recordings. The document co-citation analysis showed that a total of 27 documents were cited for more than 20 times, which comprise the key documents in metaphor research. A visualization of keywords and its cluster analysis were conducted to show the research fields and dominant topics in this field. The top 5 high-frequency keywords were language, comprehension, conceptual metaphor, discourse, and figurative language. The most prominent 5 clusters are labeled as right hemisphere, self, time, teacher education, corpus linguistics. These results revealed that metaphor research has largely focused on cognitive processing of metaphors, the influence of metaphors on self identity, the spatialization of time, and second-language learners’ metaphor ability education. Metaphor ability education belongs to one particular strand of applied linguistics, while cognitive processing of metaphors, the influence of metaphors on self identity respectively comprise interdisciplinary studies of language and cognitive science, and metaphor and pathology. The present review through CiteSpace flags the need for more investigations of CMT from more aspects or interdisciplinary studies, such as metaphor translation, metaphor in literature, metaphor and corpus linguistics, etc.

Overall, this study is the first review of linguistic research on CMT via CiteSpace tool and can provide us a sound overview of the landscape of international research on CMT. It is hoped that this study can help researchers to identify subsisting knowledge gaps and a number of future research opportunities.

## Data availability statement

The original contributions presented in this study are included in the article/supplementary material, further inquiries can be directed to the corresponding author.

## Author contributions

All authors listed have made a substantial, direct, and intellectual contribution to the work, and approved it for publication.
